# Perinatal DNA Methylation at *CDKN2A* Is Associated With Offspring Bone Mass: Findings From the Southampton Women's Survey

**DOI:** 10.1002/jbmr.3153

**Published:** 2017-05-22

**Authors:** Elizabeth M Curtis, Robert Murray, Philip Titcombe, Eloïse Cook, Rebecca Clarke‐Harris, Paula Costello, Emma Garratt, Joanna D Holbrook, Sheila Barton, Hazel Inskip, Keith M Godfrey, Christopher G Bell, Cyrus Cooper, Karen A Lillycrop, Nicholas C Harvey

**Affiliations:** ^1^ MRC Lifecourse Epidemiology Unit University of Southampton Southampton UK; ^2^ Institute of Developmental Sciences University of Southampton Southampton UK; ^3^ NIHR Southampton Biomedical Research Centre University of Southampton and University Hospital Southampton NHS Foundation Trust Southampton UK; ^4^ Singapore Institute for Clinical Sciences (SICS) A*STAR Brenner Centre for Molecular Medicine Singapore; ^5^ Centre for Biological Sciences University of Southampton Southampton UK; ^6^ NIHR Oxford Musculoskeletal Biomedical Research Unit University of Oxford Oxford UK

**Keywords:** OSTEOPOROSIS, EPIDEMIOLOGY, EPIGENETICS, DXA, CDKN2A, DEVELOPMENT

## Abstract

Poor intrauterine and childhood growth has been linked with the risk of osteoporosis in later life, a relationship that may in part be mediated through altered epigenetic regulation of genes. We previously identified a region within the promoter of the long non‐coding RNA *ANRIL* encoded by the *CDKN2A* locus, at which differential DNA methylation at birth showed correlations with offspring adiposity. Given the common lineage of adipocytes and osteoblasts, we investigated the relationship between perinatal *CDKN2A* methylation and bone mass at ages 4 and 6 years. Using sodium bisulfite pyrosequencing, we measured the methylation status of the 9 CpGs within this region in umbilical cord samples from discovery (*n* = 332) and replication (*n* = 337) cohorts of children from the Southampton Women's Survey, whose bone mass was assessed by dual‐energy X‐ray absorptiomietry (DXA; Hologic Discovery). Inverse associations were found between perinatal *CDKN2A* methylation and whole‐body minus head bone area (BA), bone mineral content (BMC), and areal bone mineral density (BMD). This was confirmed in replication and combined data sets (all *p *< 0.01), with each 10% increase in methylation being associated with a decrease in BMC of 4 to 9 g at age 4 years (*p *≤ 0.001). Relationships were similar with 6‐year bone mass. Functional investigation of the differentially methylated region in the SaOS‐2 osteosarcoma cell line showed that transcription factors bound to the identified CpGs in a methylation‐specific manner and that CpG mutagenesis modulated *ANRIL* expression. In conclusion, perinatal methylation at *CDKN2A* is associated with childhood bone development and has significance for cell function. © 2017 The Authors. *Journal of Bone and Mineral Research* Published by Wiley Periodicals Inc.

## Introduction

Although there is evidence of a substantial heritable component to bone mineral density (BMD),^1^, ^2^ there is increasing evidence that interactions between environment and genotype, leading to altered gene expression, may contribute to the overall variance in BMD.^3^ Previous population studies have shown that poor intrauterine and childhood growth are predictors of osteoporosis in later life and of adult hip fracture.^4^, ^5^ Maternal factors, such as diet before and during pregnancy, lifestyle (eg, cigarette smoking and physical activity), body composition, vitamin D status during pregnancy, and also paternal factors, such as skeletal size, have all been associated with offspring bone development.^6^, ^7^, ^8^, ^9^, ^10^ Evidence that such associations between environmental factors at critical periods of early development and later health and disease might be mediated by epigenetic mechanisms have come from the natural world and also from experimental animal studies in which altered gestational diet in rat models leads to modification of DNA methylation, gene expression, and phenotype in the offspring.^3^, ^11^, ^12^, ^13^ Previously, a candidate gene approach from our group has demonstrated that methylation at the Retinoid X Receptor–alpha (*RXRA*) promoter in umbilical cord DNA is associated with offspring bone mass in childhood,^14^ complementing our documented associations between offspring fat mass, perinatal *RXRA* methylation, and methylation at a further gene, *CDKN2A*.^15^, ^16^, ^17^

There is evidence that DNA methylation at various genes, including cyclin‐dependent kinases such as *CDKN2A*, plays a role in skeletal development, homeostasis, and bone cell activity. Thus methylation has been implicated in mechanisms of osteoblastic differentiation^18^, ^19^, ^20^ and osteoclastogenesis,^21^ together with the transition from osteoblast to osteocyte.^22^, ^23^, ^24^, ^25^ These experimental findings are complemented by data from genome‐wide methylation profiling studies in older patients, demonstrating differential methylation at genes such as the cyclin‐dependent kinase inhibitor *CKDN1C* and cyclin‐dependent kinase *CDK20* is associated with BMD.^26^, ^27^ These findings, therefore, indicate the importance of epigenetic processes in bone metabolism, particularly with regard to loci implicated in cellular differentiation, cell cycle regulation, and bone cell function.

The *CDKN2A* locus encodes two cell cycle inhibitors: p14^ARF^ and P16^INK4a^, which play roles in cellular senescence and aging. The *CDKN2A* locus also encodes the long non‐coding RNA *ANRIL* (antisense non‐coding RNA in the *INK4* locus), a 3834 bp transcript that can negatively regulate *p16^INK4a^*. Single‐nucleotide polymorphisms (SNPs) within the *CDKN2A* locus, particularly those located within *ANRIL*, have been associated with cardiovascular disease, diabetes, and frailty,^28^ and DNA methylation at this locus has recently been demonstrated to vary with age.^29^ Given our previous demonstration of links between perinatal *CDKN2A* methylation and offspring fat mass,^16^ together with the common mesenchymal origin and well‐established functional relationships between fat and bone, mediated via both mechanical and endocrine pathways,^30^ we carried out a targeted approach to examine DNA methylation at CpG sites within the *CDKN2A* gene. We examined DNA methylation across a 300 bp region within the promoter region of *ANRIL* that contained 9 CpG dinucleotides (a cytosine immediately preceding a guanine base in the 5’ to 3’ direction). We hypothesised that DNA methylation at birth would be associated with offspring bone mass in childhood. To test this, we examined DNA methylation levels in relation to BMD in a discovery cohort, then replicated our findings in a second separate cohort. We also carried out functional analysis of the region to determine its importance for local gene expression and transcription factor binding.

## Materials and Methods

### Southampton Women's Survey (SWS)

A detailed description of the SWS, a prospective mother‐offspring birth‐cohort study in Southampton, UK, has been published previously.^31^ Briefly, non‐pregnant women aged 20 to 34 years were recruited into the study (*n* = 12,583) between April 1998 and October 2002. A total of 3158 women who subsequently became pregnant and delivered a liveborn singleton infant between December 1998 and December 2007 were phenotyped in detail during pregnancy, and their offspring have been followed up during childhood.

### Offspring bone assessment

A consecutive subset of children was invited to visit the Osteoporosis Centre at Southampton General Hospital for assessment of bone mass and body composition at age 4 years. At this visit, written informed consent for a dual‐energy X‐ray absorptiometry (DXA) scan was obtained from the mother or father/guardian. The child's height (Leicester height measurer, Seca Ltd, Birmingham, UK) and weight (in underpants only, using calibrated digital scales, Seca Ltd) were measured. A whole‐body DXA scan was obtained, using a Hologic Discovery instrument (Hologic Inc., Bedford, MA, USA) in pediatric scan mode. Scans with unacceptable movement artefact were excluded. The manufacturer's coefficient of variation (CV) for the instrument was 0.75% for whole‐body bone mineral density, and the experimental CV when a spine phantom was repeatedly scanned in the same position 16 times was 0.68%. A similar bone assessment was undertaken at age 6 years. The SWS was conducted according to the guidelines laid down in the Declaration of Helsinki, and the Southampton and South West Hampshire Research Ethics Committee approved all procedures.

### Umbilical cord DNA extraction

A 5‐ to 10‐cm segment was cut from the mid portion of each cord immediately after delivery, flushed with saline to remove fetal blood, flash‐frozen in liquid nitrogen, and stored at −80°C until required for DNA isolation. Genomic DNA was isolated from frozen archived umbilical cord tissue by classical proteinase K digestion and phenol:chloroform extraction.

### Quantitative DNA methylation analysis and pyrosequencing: study and replication cohorts

**Figure 1:**
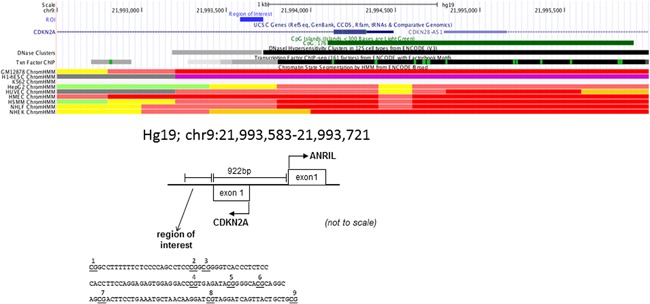
Location of CpG dinucleotides in relation to the known genes in the *CDKN2A* region. Region of interest: 21993583‐21993721 (human genome hg19/GRCh37 build). The top figure shows the UCSC‐genome annotation for this region; the lower figure comprises a clarified schematic diagram focusing on local gene layout with relevant distances marked in base pairs and the CpG dinucleotides of interest marked on an annotated primary sequence.

The region of interest within the *CDKN2A* gene locus is located within the promoter region of the non‐coding RNA *ANRIL*, transcribed from this gene locus, and contains 9 CpG dinucleotides (chr9: 21993583‐21993721) (Fig. 1). We used sodium bisulfite targeted pyrosequencing (Pyromark MD, Qiagen, Valencia, CA, USA; https://www.qiagen.com/fi/resources/technologies/pyrosequencing-resource-center/technology-overview/)^32^ to carry out in‐depth analysis of the methylation status of these 9 CpGs within the previously identified differentially methylated region of *CDKN2A* in umbilical cords. The analysis followed a discovery/replication design, with methylation status measured in an initial consecutive series of umbilical cords from SWS deliveries (Discovery cohort), on whom childhood DXA assessment had been undertaken. Subsequently, when DXA measures had become available on a further separate subset of SWS children, methylation at these CpG sites was measured in a second, consecutive and independent series of SWS deliveries (Replication cohort). Inter‐ and intraplate controls were added to each plate as a control for inter‐ and intraplate variability, and 0% and 100% methylation controls were run to ensure that the full range of methylation could be detected. The summary statistics for methylation (minimum, maximum, quartile 1, median, quartile 3), plus mean and SD, together with the genomic coordinates for the *CDKN2A* CpG sites are shown in Supplemental Table S1. Details of the studies of functional validation in osteosarcoma SaOS‐2 cells are presented in the Supplemental Material and summarized in the relevant paragraphs of the Results section.

### Statistical analysis

Statistical analysis was undertaken using Stata (StataCorp, College Station, TX, USA; versions 14.0/14.1/14.2). The data were checked to ensure that the assumptions of linear regression were met. Regression models were built using the offspring bone indices measured by DXA (at either 4 or 6 years) as the outcome and CpG methylation as the predictor. Results are presented as the number of observations in each regression and regression coefficients (β) with their associated *p* values and 95% confidence intervals. β coefficients represent the change in the bone outcome per 10% change in methylation at each CpG site. This analysis was initially performed on the SWS Discovery cohort, repeated in the SWS Replication cohort, and then analysis was performed on the two cohorts combined, described as the SWS Combined cohort, in order to maximize statistical power for further multivariate analyses. All models were adjusted for the child's sex and age at DXA scan, except for at 4 years where an adjustment for age was not necessary because of the narrow age range at which the DXA scans were performed. Where analysis was performed on the Combined cohort, an indicator representing cohort was included as a covariate in the model to account for batch effect. In multivariate models, we accounted for potential confounding factors previously found to be associated with offspring bone development (mother's pre‐pregnancy height, maternal parity, late pregnancy walking speed, late pregnancy maternal smoking, and late pregnancy triceps skinfold thickness). In further analyses, we explored the effect of the covariates potentially on the causal pathway, including offspring birthweight, and childhood height, weight, lean mass, and fat mass.

Recognizing that there was likely to be co‐linearity between the individual exposures and between the DXA indices, and given the relatively small number of tests in our analysis compared with larger‐scale genome‐wide association studies, for which methods such as Bonferroni or the Benjamini‐Hochberg/False Discovery Rate corrections for multiple testing would be appropriate,^33^ we undertook a data reduction approach by investigating clustering of the CpG methylation. Importantly, there is evidence that where clusters of differential CpGs can be identified, they are more likely to be functional than are individual CpG changes.^34^ By investigating the correlation between methylation at each of the individual CpG sites and calculating the median absolute deviation (MAD) from the median for each site, we grouped the CpG sites into 4 clusters (CpG1‐2, 3, 4‐7, 8‐9), with each cluster represented by the site with the highest MAD score (ie, the site with the greatest variability within the cluster), that is, CpG sites 2, 3, 7, and 9, respectively (summarized in Supplemental Fig. S2). For completeness, we also used the Simes modification of the Benjamini‐Hochberg method to undertake a *p* value correction on the analyses in the Combined data set, using the Stata “qqvalue” command, which is similar to the “p.adjust” command in R.^34^ These are presented as *q* values in Supplemental Table S7.

## Results

### Characteristics of the subjects

There were 332 mother and child pairs with methylation measurements at the 9 CpG sites within the *CDKN2A* locus and DXA data at either 4 years or 6 years of age in the SWS Discovery cohort, 374 in the SWS Replication cohort, and 706 in the SWS Combined cohort, in which the Study and Replication cohorts were combined. Tables 1 and 2 summarize the characteristics of the mothers and children in the Discovery, Replication, and Combined cohorts. Compared with mothers in the SWS as a whole, mothers in the final Combined cohort were slightly taller (*p* = 0.004), less likely to smoke during pregnancy (*p* = 0.049), and had higher educational qualifications (*p *< 0.001) and higher socioeconomic status (*p *< 0.001).

**Table 1:** Characteristics of the SWS Mothers in the Study Cohort (SWS 1st 400), Replication Cohort (SWS 2nd 400), and Combined Cohort (SWS 800)

Characteristic	% or median (5th, 95th percentile) for SWS Study cohort *mean (SD) *n *= 332	% or median (5th, 95th percentile) for SWS Replication cohort *mean (SD) *n *= 374	% or median (5th, 95th percentile) for combined SWS cohort *mean (SD) *n *= 706
Woman's age at birth of child (years)	30.44 (3.5)*	31.23 (3.6)*	30.86 (3.6)*
Body mass index	24.29 (19.8, 34.6)	24.10 (19.6, 34.4)	24.19 (19.7, 34.6)
Maternal height (cm)	163.72 (6.7)*	163.91 (6.2)*	163.82 (6.4)*
Maternal weight (kg)	66.0 (52.2, 93.8)	64.8 (51.9, 96.3)	65.7 (51.9, 94.3)
Late pregnancy mid upper arm circumference (cm)	30.04 (3.5)*	30.37 (3.7)*	30.22 (3.6)*
Smoking (during pregnancy)	13.64%	14.21%	13.94%
Educational qualifications			
None	0.90 %	2.43%	1.71%
CSE	8.73%	8.89%	8.82%
O levels	26.81%	27.76%	27.31%
A levels	27.11%	35.04%	31.29%
HND	9.04%	6.47%	7.68%
Degree	27.41%	19.41%	23.19%
Social class			
Professional	6.75%	4.62%	5.62%
Management and technical	42.64%	39.13%	40.78%
Skilled non‐manual	32.21%	37.50%	35.01%
Skilled manual	6.75%	7.07%	6.92%
Partly skilled	10.74%	10.33%	10.52%
Unskilled	0.92%	1.36%	1.15%
Late pregnancy walking speed			
Very slow	15.26%	16.76%	16.06%
Stroll at an easy pace	53.58%	52.43%	52.97%
Normal speed	26.48%	23.78%	25.04%
Fairly brisk	4.36%	6.76%	5.64%
Fast	0.31%	0.27%	0.29%

**Table 2:** Characteristics of the SWS Children in the Discovery, Replication, and Combined Cohorts

	% or median (5th, 95th percentile) *mean (SD)		
Characteristic	Discovery cohort *n *= 332	Replication cohort *n *= 374	Combined cohort *n *= 706
Female	47.89%	51.34%	49.72%
Birth order			
1st	47.59%	51.07%	49.43%
2nd	39.46%	36.10%	37.68%
3rd or lower	12.95%	12.83%	12.89%
Birth weight (kg)	3.46 (0.5)*	3.53 (0.5)*	3.50 (0.5)*
Gestational age (weeks)	40.00 (36.6, 41.9)	40.14 (37.1, 41.9)	40.12 (37.0, 41.9)
Placental weight (g)	467.95 (99.3)*	479.09 (104.2)*	473.94 (102.0)*
4‐Year child DXA bone indices^a^			
4‐Year age at DXA scan	4.1 (4.0, 4.2)	4.1 (4.0, 4.2)	4.1 (4.0, 4.2)
Total BMC (g)	374.6 (45.0)*	371.8 (45.3)*	373.2 (45.1)*
Total BMD (g/cm^2^)	0.37 (0.05)*	0.37 (0.05)*	0.37 (0.05)*
Total BA (cm^2^)	0.49 (0.04)*	0.49 (0.04)*	0.49 (0.04)*
4‐Year total fat mass^a^ (kg)	4.1 (2.9, 6.7)	4.1 (3.0, 6.7)	4.1 (2.9, 6.7)
4‐Year total lean mass^a^ (kg)	9.86 (1.3)*	9.73 (1.3)*	9.79 (1.3)*
6‐Year child bone DXA indices^b^			
6‐Year age at DXA scan	6.6 (6.3, 7.0)	7.0 (6.5, 7.5)	6.8 (6.3, 7.4)
Total BA (cm^2^)	908.06 (64.2)*	913.18 (62.4)*	910.65 (63.3)*
Total BMC (g)	547.0 (74.1)*	551.6 (77.1)*	549.3 (75.6)*
Total BMD (g/cm^2^)	0.60 (0.05)*	0.60 (0.05)*	0.60 (0.05)*

^a^ Whole body minus head site, adjusted for sex.

^b^ Whole body minus head site, adjusted for sex and age.

### *CDKN2ACDKN2A* methylation is associated with offspring bone size, mineralization, and areal density

**Figure 2:**
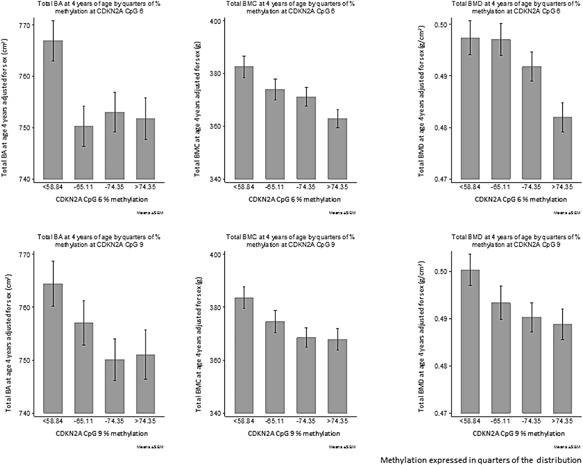
*CDKN2A* CpG methylation in relation to bone mineral outcomes. Percentage methylation at *CDKN2A* CpG 6 (left) and CpG 9 (right) expressed in quarters of the distribution in umbilical cord tissue, and offspring total bone area (whole body minus head, cm^2^), bone mineral content (g), and areal bone mineral density (g/cm^2^) at age 4 years.

Percentage methylation at the 9 CpG sites varied greatly, from 17.1% to 99.6% (Supplemental Table S1). Tables 3 and 4 show that there were inverse associations between CpG methylation at sites 3, 7, and 9 (with CpGs 7 and 9 representing the clusters CpG4‐7 and CpG8‐9) and offspring whole‐body minus head bone indices at 4 years. Because the relationships were similar in both cohorts, we used the Combined Cohort for all remaining analysis (Table 5, Fig. 2). Thus, in the combined cohort, both unadjusted and after adjustment for batch effect, mother's late pregnancy (LP) walking speed, LP smoking, prepregnancy height, LP triceps skinfold thickness, and parity, there were strongly statistically significant inverse associations between CpG methylation at CpG sites 3, 7, and 9 (but not CpG2) with the bone indices at 4 years (Table 6). Similar inverse associations were observed at 6 years (Supplemental Table S3). At other CpG sites, inverse but non‐statistically significant associations were observed. The effect sizes were similar across different CpG sites and the two different ages; for example, for every 10% increase in methylation at CpG9, there was a 9.1 g decrease in whole‐body minus head BMC at age 4 years and a 10.2 g decrease at age 6 years. In further analyses aimed at identifying potential mediators of the methylation‐bone relationship, the CpG‐bone associations remained robust after inclusion of child's whole‐body lean mass or fat mass or child's birthweight in the multivariate models (Supplemental Table S4). CpG‐bone associations for total BMC and total BMD also persisted after adjustment for the child's height at DXA (Supplemental Table S5), though associations between CpG methylation and total BA were attenuated. Adjustment for body weight markedly attenuated the associations to below statistical significance.

**Table 3:** *CDKN2A* CpG Methylation and Bone Mineral Outcomes at Age 4 Years in the SWS Discovery Cohort (Relationship Between Methylation at the 9 CpG Sites Within the *CDKN2A* Region of Interest and Total Bone Area, Bone Mineral Content, and Bone Mineral Density in the 4‐Year‐Old Children [Whole Body Minus Head])

	Discovery	Total BA (cm^2^)			Total BMC (g)			Total BMD (g/cm^2^)			
CpG cluster	*n*	β	*p* Value	95% CI	β	*p* Value	95% CI	β	*p* Value	95% CI	
1–2	254	−3.07	0.324	(–9.18, 3.05)	−3.06	0.297	(−8.84, 2.71)	−0.0018	0.442	(−0.0062, 0.0027)	
3	229	−3.32	0.428	(–11.57, 4.92)	−4.64	0.243	(−12.46, 3.17)	−0.0037	0.236	(−0.0097, 0.0024)	
4–7	288	−6.94	**0.037**	(–13.44, –0.44)	−6.23	**0.05**	(−12.44, –0.01)	−0.0033	0.195	(−0.0083, 0.0017)	
8–9	262	−10.85	**0.005**	(–18.47, –3.23)	−10.27	**0.005**	(−17.47, –3.07)	−0.006	**0.045**	(−0.0118, −0.0001)	

Associations adjusted for sex. β coefficients and 95% CIs have been multiplied by 10 and therefore represent the change associated with a 10% increase in methylation. *p* values <0.05 are in bold.

**Table 4:** *CDKN2A* CpG Methylation and Bone Outcomes at Age 4 Years in the SWS Replication Cohort (Relationship Between Methylation at the 9 CpG Sites Within the *CDKN2A* Region of Interest and Total Bone Area, Bone Mineral Content, and Bone Mineral Density in the 4‐Year‐Old Children [Whole Body Minus Head])

	Replication	Total BA (cm^2^)			Total BMC (g)			Total BMD (g/cm^2^)			
CpG cluster	*n*	β	*p* Value	95% CI	β	*p* Value	95% CI	β	*p* Value	95% CI	
1–2	245	−2.58	0.399	(−8.61, 3.44)	−4.94	0.09	(−10.66, 0.77)	−0.0046	0.051	(−0.0092, 0.000002)	
3	193	−9.8	**0.016**	(−17.74, −1.86)	−10.22	**0.008**	(−17.76, −2.69)	−0.007	**0.027**	(−0.0131, −0.0008)	
4–7	267	−6.93	**0.025**	(−12.96, −0.89)	−9.9	**0.001**	(−15.67, −4.14)	−0.0081	**0.001**	(−0.0128, −0.0035)	
8–9	216	−6.39	0.056	(−12.95, 0.17)	−8.79	**0.008**	(−15.24, −2.35)	−0.007	**0.009**	(−0.0122, −0.0018)	

Associations adjusted for sex. β coefficients and 95% CIs have been multiplied by 10 and therefore represent the change associated with a 10% increase in methylation. *p* values <0.05 are in bold.

**Table 5:** *CDKN2A* CpG Methylation and Bone Mineral Outcomes at Age 4 Years in the SWS Combined Cohort (Relationship Between Methylation at the 9 CpG Sites Within the *CDKN2A* Region of Interest and Total Bone Area, Bone Mineral Content, and Bone Mineral Density in the 4‐Year‐Old Children [Whole Body Minus Head])

	Combined	Total BA (cm^2^)			Total BMC (g)			Total BMD (g/cm^2^)			
CpG cluster	*n*	β	*p* Value	95% CI	β	*p* Value	95% CI	β	*p* Value	95% CI	
1–2	499	−2.83	0.1945	(−7.11, 1.45)	−3.99	0.0538	(−8.04, 0.07)	−0.0031	0.0543	(−0.0064, 0.0001)	
3	422	−6.52	**0.0255**	(−12.25, −0.80)	−7.4	**0.0076**	(−12.83, −1.98)	−0.0053	**0.0162**	(−0.0096, −0.0010)	
4–7	555	−6.93	**0.0021**	(−11.34, −2.52)	−8.18	**0.0002**	(–12.40, −3.96)	−0.0059	**0.0008**	(−0.0093, −0.0025)	
8–9	478	−8.28	**0.0011**	(−13.23, −3.32)	−9.42	**0.0001**	(−14.18, −4.65)	−0.0066	**0.0009**	(−0.0104, −0.0027)	

Associations adjusted for sex and batch. β coefficients and 95% CIs have been multiplied by 10 and therefore represent the change associated with a 10% increase in methylation. *p* values < 0.05 are in bold.

**Table 6:** *CDKN2A* CpG Methylation and Bone Mineral Outcomes at Age 4 Years Accounting for Maternal Factors in the Combined SWS Cohort (Relationships Between Methylation at the Clustered CpG Sites Within the *CDKN2A* Region of Interest and Whole‐Body Minus Head Bone Area, Bone Mineral Content, and Bone Mineral Density in the 4‐Year‐Old Children)

	Combined	Total BA (cm^2^)			Total BMC (g)			Total BMD (g/cm^2^)			
CpG cluster	*n*	β	*p* Value	95% CI	β	*p* Value	95% CI	β	*p* Value	95% CI	
1–2	484	−3	0.1628	(−7.21, 1.22)	−4.11	**0.0407**	(−8.05, −0.17)	−0.0032	**0.0429**	(−0.0064, −0.0001)	
3	408	−5.93	**0.0431**	(−11.68, −0.18)	−7.01	**0.0099**	(−12.33, −1.69)	−0.0052	**0.0154**	(−0.0094, −0.0010)	
4–7	538	−5.85	**0.0084**	(−10.20, 1.51)	−7.17	**0.0007**	(−11.30, −3.04)	−0.0053	**0.0021**	(−0.0086, −0.0019)	
8–9	461	−8.26	**0.0009**	(−13.11, −3.41)	−9.11	**0.0002**	(−13.79, −4.43)	−0.0062	**0.0016**	(−0.0100, −0.0024)	

Adjusted for batch, child's sex, mother's late pregnancy (LP) walking speed, LP smoking, prepregnancy height, LP triceps skinfold thickness, and parity. β coefficients and 95% CIs have been multiplied by 10 and therefore represent the change associated with a 10% increase in methylation. *p* values <0.05 are in bold.

### Functional analysis of the differentially methylated region of *CDKN2ACDKN2A* in osteosarcoma cells

**Figure 3:**
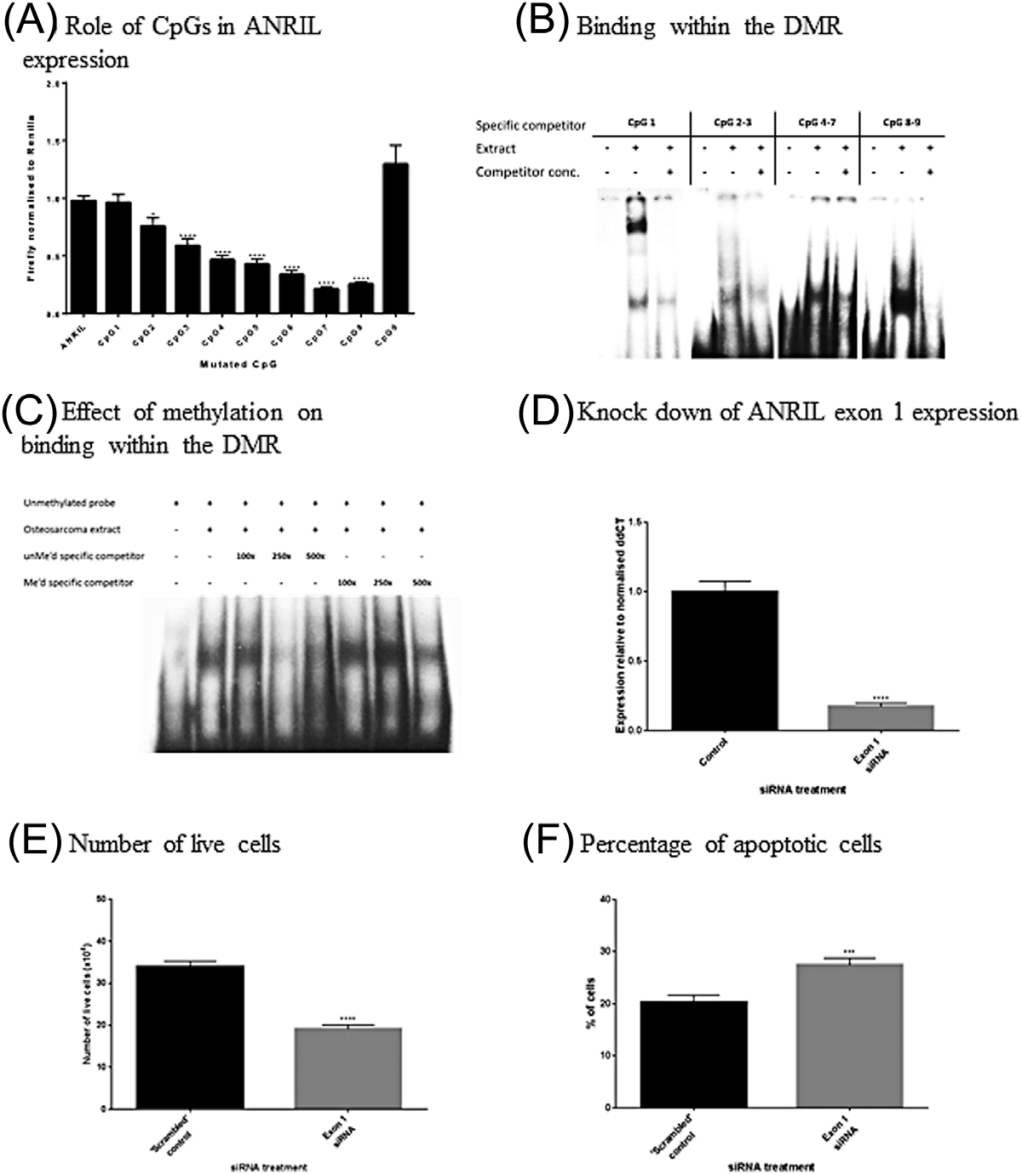
CpG sites within the DMR are important for the transcriptional regulation of the *CDKN2A* cluster. (*A*) Role of CpGs in expression SaOS‐2: cells were transiently transfected with *ANRIL* promoter constructs in a pGL3‐basic reporter background. CMV‐renilla was co‐transfected for normalization. (*B*) Electrophoretic mobility shift assays using nuclear extract from osteosarcoma cell line SaOS‐2 to examine nuclear protein binding to different regions within the DMR. A lane without protein extract was used as negative control, and non‐radiolabeled probe at 500× was used as a specific competitor to examine if binding is specific to that region of the DMR. (*C*) EMSAs with unmethylated‐ and methylated‐specific competitor were used in increasing concentrations of 100‐, 250‐, and 500‐fold to determine if methylation has an effect on protein binding to this region. (*D*) Transient transfection of SaOS‐2 cells with 10 nM siRNA against exon 1 of *ANRIL* successfully knocked down exon 1 expression in these cells. (*E*) SaOS‐2 cells were treated for 72 hours with “scrambled” siRNA (negative control) and siRNA against exon 1 of *ANRIL*. Cells were trypsinized and number of live cells counted for each siRNA treatment. (*F*) DNA from SaOS‐2 cells treated with “scrambled” and *ANRIL* exon 1 siRNAs was stained with propidium iodide and FACS analysis used to determine percentage of cells undergoing apoptosis. Figures represent pooled results from three independent experiments (*p < 0.05, **p < 0.01, ***p < 0.001, ****p < 0.0001).

Because the 9 CpG sites are located within the promoter region of the long non‐coding RNA *ANRIL*, we next investigated whether the CpG sites might be important for the expression of *ANRIL*. The promoter region of *ANRIL* (–1281 bp to +20 bp relative to TSS) was fused upstream of the luciferase reporter gene in pGL3Basic. The 9 CpG sites were then individually mutated (CpG>TpG) and each construct transfected into the human osteosarcoma cell line SaOS2. Mutation of CpG2‐8 led to a decrease in *ANRIL* promoter activity (*p *≤ 0.05), whereas mutation of CpG1 and CpG9 had no effect on *ANRIL* promoter activity (Fig. 3 *A*). To determine whether methylation of the CpG sites associated with later bone outcomes might affect transcription factor binding to this region, electrophoretic mobility shift assays (EMSA) were undertaken (full details in the Supplemental information). Incubation of nuclear extracts from SaOS‐2 cells with oligonucleotides covering CpG1, CpG2‐3, CpG4‐7, and CpG8‐9 showed strong specific binding of protein complexes to the oligonucleotides containing CpG1 and CpG8‐9, with weaker binding also observed to CpG2‐3 and CpG4‐7 (Fig. 3 *B*, *C*).

To test whether DNA methylation affected protein binding, the CpG8‐9 oligonucleotide was incubated with nuclear extracts from SaOS‐2 cells with a 50‐, 100‐, and 500‐fold excess of unmethylated‐ or methylated‐specific competitor, containing a methylated cytosine at CpGs 8 and 9.^35^ Although binding to the probe was markedly reduced in the presence of a 100‐fold excess of the unmethylated‐specific competitor, there was little reduction in binding upon the addition of a 100‐fold excess of cold competitor containing the methylated CpGs. When a methylated probe was used, the unmethylated competitor again competed out binding much more effectively than the methylated competitor. Comparison of binding to both the unmethylated and methylated probes also showed stronger binding to the unmethylated probe (Supplemental Fig. S2).

To identify which transcription factors bind to this region of *CDKN2A*, DNA consensus sequences for 80 common transcription factors^35^ were used to examine binding within the CpG8‐9 region. Transcription factor consensus sequences for interferon‐gamma activated site (GAS) and SMAD3/4 competed‐out binding of radiolabeled probe to the CpG8‐9 region in osteosarcoma cell line extract (Supplemental Fig. S2*B*). Consistent with these findings, in silico analysis of ENCODE ChromHMM data^36^ revealed that this region is enriched for both promoter and enhancer activity across multiple cell types, as well as overlapping DNase I hypersensitive sites, suggesting that the CpG sites examined in this article lie within a key regulatory region of *CDKN2A* (Fig. 1, Supplemental Fig. S2*C*).

Having determined that the CpGs sites associated with later bone outcomes may have functional relevance for *ANRIL* promoter activity in osteosarcoma cells, we next investigated the effect of perturbing *ANRIL* expression in SaOS‐2 cells. SaOS‐2 cells were transfected with siRNA directed against *ANRIL* 28 and the effect on cell growth and apoptosis assessed. Primer sequences are summarized in Supplemental Table S2. Transfection of the *ANRIL* siRNA caused a fivefold decrease in *ANRIL* expression (Fig. 3 *D*) and decreased the number of live cells by 0.3‐fold (*p *≤ 0.001) (Fig. 3 *E*), while increasing the number of cells undergoing apoptosis (FACS) (*p *≤ 0.001) (Fig. 3 *F*).

## Discussion

We found that lower perinatal methylation of specific CpG dinucleotides within the *CDKN2A* gene locus is associated with higher total whole‐body minus head BA, BMC, and areal BMD at 4 and 6 years of age. Furthermore, we demonstrated that these CpG sites may play a role in modulating the level of expression of the long non‐coding RNA ANRIL, which regulates cell survival, suggesting that these relationships have functional relevance.

To our knowledge, this is the first time that associations between *CDKN2A* methylation and childhood bone development and the related consequences for cell survival and gene expression have been demonstrated. We have previously published data relating methylation at the Retinoid X Receptor–alpha (*RXRA*) to childhood bone mineral content corrected for body size^14^ and associations at this and other sites in relation to offspring adiposity.^15^ The *CDKN2A* (*INK4A‐ARF*) gene locus encodes two potent inhibitors of cell growth: *p14^ARF^* (alternative reading frame relative to p16) and *p16^INK4a^* (inhibitor of cyclin‐dependent kinase 4). Recently, both *p16^INK4a^* and *p14^ARF^* have been shown to play a role in driving cellular senescence and aging.^37^, ^38^ Consistent with a role for the *CDKN2A* locus in aging, GWAS studies have shown that SNPs in a region spanning 160 kb around the *CDKN2A* locus, with the majority located within *ANRIL*, were associated with increased susceptibility to frailty, coronary artery disease, myocardial infarction, type 2 diabetes, and late‐onset Alzheimer disease.^28^, ^39^, ^40^, ^41^, ^42^, ^43^, ^44^, ^45^ Although the majority of these SNPs are located toward the 3’ end of the *ANRIL* coding region, more than 100 Kb from the differentially methylated region (DMR) identified in this study, these SNP associations and recent evidence linking methylation at *CDKN2A* with aging^29^ do highlight the importance of the *CDKN2A* locus in general and *ANRIL* in particular in altered susceptibility to aging‐related diseases.

There is little evidence in the literature for the specific involvement of the *CDKN2A* locus in bone metabolism, although *p16^INK4a^* expression has been linked to altered osteoblast morphology and senescence in animal models,^46^, ^47^ and one study identified transitional hypomethylation of *CDKN2A* in human bone marrow stromal cells in their differentiation toward an osteoblastic lineage.^18^ However, the importance of cyclin‐dependent kinases and their inhibitors has been demonstrated by studies of the osteogenic differentiation of adipose‐derived mesenchymal stem cells, in which the promoters of *RUNX2*, *osteocalcin*, and *osterix* genes are actively demethylated in a process dependent upon growth arrest and DNA‐damage‐inducible protein, GADD45, which is known to interact with both *CDK1* and *CDKN1A*.^19^, ^20^ Genome‐wide methylation profiling studies in older patients comparing individuals with low versus normal BMD have also suggested early life influences on bone quality in older age.^27^ In a genome‐wide association study, genes encoding cyclin‐dependent kinase inhibitor *CKDN1C* and cyclin‐dependent kinase *CDK20* have been found to be differentially methylated in ex vivo bone samples from patients who have experienced a low‐trauma hip fracture compared with those from patients undergoing elective arthroplasty for osteoarthritis.^26^ Furthermore, DMRs enriched in genes associated with cell differentiation and skeletal embryogenesis, including those in the homeobox superfamily, were identified, supporting a developmental component to these conditions.

There are several possible explanations for our findings: first, given that we have also observed inverse associations between *CDKN2A* methylation and offspring adiposity,^16^ the relationships with bone might have been mediated through fat or lean mass. However, when we adjusted for DXA whole‐body fat or lean mass in the multivariate models, the *CDKN2A*‐bone relationships remained robust. Second, it is possible that *CDKN2A* methylation and childhood bone mass are both influenced by a common factor during intrauterine life. In this situation, one might expect that the *CDKN2A*‐bone relationships would be attenuated through adjustment for birthweight, but we found that statistically significant relationships remained (Supplemental Table S4), although were markedly attenuated by inclusion of current weight in the model. This latter observation is difficult to interpret owing to the high correlation between body weight and bone indices, but the persisting associations between perinatal *CDKN2A* methylation and bone indices after adjustment for childhood height suggests that they are not mediated by skeletal size alone. Finally, it is possible that *CDKN2A* methylation in umbilical cord tissue does have a causal relationship with bone development, particularly given the common mesenchymal origin of elements of both tissues, such that the alterations to methylation detected in umbilical cord tissue are consistent with changes in bone tissue or that the umbilical cord methylation marks influence other processes, which in turn influence bone development. It seems intuitively reasonable that a resetting of gene expression, via epigenetic marking, might have a long‐term influence on skeletal growth. Our epidemiological findings are supported by our in vitro investigations, in which we demonstrated the functional importance of these CpG dinucleotides for ANRIL expression and that DNA methylation affects protein binding in the region, results that indicate the functional relevance of the methylation signals, including potential roles for specific transcription factors. Importantly, although elucidation of the causal (or not) nature of the associations is essential for mechanistic understanding and identification of potential therapeutic targets, it is less important in terms of risk stratification and identification of those individuals potentially at increased risk of low bone density in old age.

We used a prospective cohort with detailed characterization of mothers and children, using the gold standard DXA technique to assess bone mass. There are, however, several limitations to our study. First, although we previously excluded the presence of a SNP at the CpG sites of interest by sequencing,^17^ it is not possible to exclude a genetic *trans*‐effect of distant SNPs that could influence both DNA methylation of a particular sequence and child's phenotype. Second, we analyzed methylation in cells from whole umbilical cord; although it is possible that the differential methylation we observed arose from variation in the proportions of different component cells (for example, fibroblasts, epithelial cells) in individual samples, our results were consistent across both our Discovery and Replication set. Also, our studies show similar methylation in different umbilical cord cell types (unpublished). Furthermore, any unaccounted cell‐heterogeneity that is being observed as epigenetic change may represent proportional differences that are related to the phenotypic outcomes.^48^ Third, we were not able to assess *CDKN2A* DNA methylation in bone itself because of the difficulties in obtaining such samples from children and have therefore used DNA methylation in the cord as a proxy. There are a growing number of studies that have found that the methylation status of CpGs in peripheral tissues such as blood correlates with that of internal tissues.^49^, ^50^ In addition, both bone and cord tissue are derived from the mesoderm and share mesenchymal cell origins; mesenchymal stem cells differentiate into osteoblasts, playing a role in bone formation both in the embryo^51^ and in the adult, in fracture and repair mechanisms.^52^ Fourth, we could not examine whether the changes in methylation were associated with differences in the expression of transcripts from this locus, however; although methylation changes may be tissue‐independent, altered gene expression patterns would most likely be cell type–dependent and reliant upon cell‐specific transcription factor expression whose function is then modulated by altered access to the underlying DNA as a result of altered methylation patterns. Fifth, measurement of bone mineral in children is hampered by their low absolute BMC. However, we used specific pediatric software, and studies of DXA indices compared with ashed mineral content in piglets have confirmed the accuracy of the technique.^53^ Sixth, the study cohort was a subset of the SWS, but mothers whose children underwent DXA scanning and those whose children did not were broadly similar: The former were on average slightly older and smoked slightly less. There is no reason to suppose, however, that relationships between *CDKN2A* promoter methylation in umbilical cord and childhood bone mineral accrual would differ between these two groups. Seventh, it should be noted that our Study and Replication cohorts were both located in Southampton, UK, forming part of the Southampton Women's Survey. Ideally, these results would go on to be replicated in a geographically separate and possibly ethnically diverse cohort. Finally, the use of DXA does not allow measurement of true volumetric bone density, thus making it difficult to be certain about differential determinants of skeletal size and volumetric density.

In conclusion, we have demonstrated that increasing methylation at CpG sites within *CDKN2A* in umbilical cord is associated with decreased bone size, mineral content, and mineral density in childhood, that these CpG sites are functionally important for local gene expression, and that DNA methylation alters transcription factor binding within the region. These findings yield mechanistic insights into the early determinants of skeletal growth and may identify novel biomarkers for future adverse bone development.

## Disclosures

All authors state that they have no conflicts of interest.

## Supporting information

Supporting Data S1.
