# Supplementary material for: Perinatal DNA Methylation at CDKN2A Is Associated With Offspring Bone Mass: Findings From the Southampton Women's Survey
Source: J Bone Miner Res. 2017 May 22;32(10):2030–40. doi: 10.1002/jbmr.3153 (PMC5528139; doi:10.1002/jbmr.3153)
Supplement: Supplementary file 1 — Supporting Data S1. [file JBMR-32-2030-s001.docx]

**ONLINE SUPPLEMENTARY INFORMATION**

**Laboratory Methods**

(Primer sequences are summarised in Online Supplementary Table 2)

*Plasmid construction*

PCR was used to amplify the ANRIL promoter: two sets of PCR primers were used, the first set amplified the ANRIL promoter (-926 to +20 relative to ANRIL TSS). A HindIII restriction site was added to the forward primer, and an NcoI restriction site to the reverse primer for cloning into pGL Basic (Promega, UK) to create pGL -951. The second PCR primer pair amplified a region containing the region of differential methylated identified by the previous genome wide study, immediately adjacent to the already cloned ANRIL promoter region (-1281 to -925). A KpnI restriction site was added to the forward primer, and a HindIII restriction site to the reverse primer for cloning into pGL -951. The completed plasmid, pGL ANRIL, contained the full genomic sequence from -1281 to +20 of the ANRIL promoter . All PCR amplification was carried out using Hot Star High Fidelity DNA polymerase (QIAGEN). The primers used are listed in Supplementary table x. The base pair sequence of the cloned region was confirmed by sequencing (GATC, Germany).

*Site directed mutagenesis*

Mutation of the individual 9 CpG sites within the promoter of ANRIL was performed using QuikChange PCR mutagenesis. Primers were designed using QuikChange Primer Design, and PCR carried out using the SequalPrep™ Long PCR Kit (Life Technologies A10498) following manufacturers guidelines. Mutants were confirmed by sequencing (GATC, Germany).

*Cell culture and transfection*

The human osteosarcoma cell line, SaOS-2, was cultured in DMEM/Ham’s F12 with 10% Fetal Bovine Serum and 1% Penicillin/Streptomycin. Cells were cultured in 96 well plates for 24hrs prior to transfection. 100ng of prepared plasmid DNA was transfected per well with six replicates per transfection. The pGL CMV Renilla plasmid (Promega UK) was co-transfected as a control. Transfections were carried out using FuGENE HD (Switchgear Genomics USA) following manufacturers’ guidelines. Transfected cells were cultured for 48hrs prior to addition of lysis buffer. Luciferase assays were carried out using the Dual-Luciferase® Reporter Assay System (Promega UK), on a VarioSkan Flash Luminometer (ThermoScientific). Firefly luciferase levels were normalised to renilla luciferase controls.

*Electrophoretic mobility shift assays (EMSAs)*

Nuclear extracts were prepared from human osteosarcoma cell line Saos2. Cell pellets were re-suspended in 800ul of lysis buffer (10mM Hepes, pH 7.9, 10mM KCl, 0.1mM EDTA, 1mM DTT, 0.5mM PMSF, 1mM leupeptin) and incubated on ice for 10 min. Then 50ul of NP-40 was added, the cells vortexed for 10 seconds and spun at 11,000g for 30 seconds. The supernatant was discarded and the pellet containing nuclei re-suspended in 50ul of nuclear extraction buffer (20mM HEPES, Ph 7.9, 0.4M NaCl, 1mM EDTA, 1mM DTT, 1mM PMSF). This was then incubated on ice for 30 min and nuclear debris pelleted at 4C for 5 minutes at 11,000g. The protein concentration of nuclear extracts was determined with the BCA protein assay kit (Pierce) using the manufacturer’s instructions. The nuclear extracts were stored at -80C until use. For EMSAs, DNA oligonucleotides were purchased from Biomers (biomers.net GmbH, Germany) and methylated oligonucleotides from Eurofins UK Ltd (Grimsby, UK) (Supplementary Table 2). Single-stranded oligonucleotide probes were annealed by heating equimolar amounts of complementary strands to 95 ^o^C for 5 minutes and slowly cooling the reaction mixture to room temperature. EMSA reactions were carried out in the presence of 2pmol of the non-specific DNA Poly (dI*dC), binding buffer (final concentration 2.5 % glycerol, 0.05 % NP-40, 5mM MgClg), 5µg nuclear extract, and up to 500-fold excess unradiolabelled competitor. Samples were incubated on ice for 10 minutes prior to addition of probe radiolabelled with γ-32P ATP. Samples were incubated at room temperature for 10 minutes then resolved on pre-run 4% non-denaturing polyacrylamide gels in 0.5X Tris-borate-EDTA for 1 hour at 100V. Gels were subsequently dried and exposed to photographic film for 24hrs.

*ANRIL siRNA-mediated silencing*

To knockdown ANRIL expression, a siRNA directed against exon 1 of ANRIL (sequence (5’-3’): GAAUGUCAGUUUUGAACUAtt was used, this siRNA has previously been used to knockdown ANRIL in vascular smooth muscle cells ([1](#_ENREF_1),[2](#_ENREF_2)). Silencer® Negative Control #1siRNA (Ambion) was used as a negative control and AllStars Hs Cell Death Control siRNA (Qiagen) was used as a positive control. SaOS-2 cells were cultured in 24-well plates and transfected with 1.5μL/well of Lipofectamine RNAiMAX with final concentrations of 10nM/well of each siRNAs. Cells were trypsinised and counted after 72hr treatment with siRNA.

*FACS analysis*

SaOS-2 cells that had been transfected with ‘scrambled’ siRNA and siRNA against exon 1 of ANRIL for 72hrs were collected by trypsinisation, pelleted at 1000rpm for 5 minutes. Cells were then washed with 1x PBS, pelleted using same conditions and re-suspended in 70% ice cold ethanol after removal of 1x PBS. After 2hrs at 4^o^C, cells were pelleted again (1000rpm; 5 minutes), ethanol aspirated and cells re-suspended in 1x PBS before spinning again at 1000rpm for 5 minutes. PBS was then removed from samples and cells re-suspended in 200μL propidium iodide staining mixture (189μL 1x PBS, 10μL 20x propidium iodide and 1μL 200x RNase A per sample). Samples were left in dark at 37^o^C for 30 minutes, after which they were transferred on to ice and analysed on the flow cytometer. 5000 events were measured for forward and side scatter on FACScalibur® flow cytometer with FL2 channel (258nm λ) and analysed using Cellquest^TM^ software from Applied Biosystems. Gating was used to select for live and dead cells and to outline each phase of the cell cycle. This allowed for determination of the percentage of gated cells in each phase of the cell cycle.

*RT-qPCR*

RNA was extracted from transfected SaOS-2 cells using TRI Reagent (Sigma) as per manufacturer’s instructions and M-MLV Reverse Transcriptase kit (Promega) used for cDNA conversion as per manufacturer’s instructions. ANRIL exon 1 expression was determined through TaqMan® non-coding RNA assay (Applied Biosystems) that covered ANRIL exon1/2 boundary (Hs04259476_m1). TaqMan gene expression assays were also used to determine RUNX2 (Hs00231692_m1), ALP (Hs01029144_m1) and p16^INK4A^ (Hs02902543_mH) expression. RPL13A was used as a housekeeping gene. Each reaction contained 100ng cDNA, 1μL 20x TaqMan assay, 10μL TaqMan Gene Expression Master Mix (Applied Biosystems) and 5μL RNase-free water and were run in triplicate. PCR cycling conditions were polymerase activation step of 95^o^C for 10 minutes, then 40 cycles of 95^o^C 15 seconds and annealing/extension at 60^oC^ for 1 minute.

**Online Supplementary Table 1:** Percentage DNA methylation at CDKN2A in umbilical cord tissue of offspring in Combined cohort. DMR: 21993583-21993721 (Human genome hg19/GRCh37 build).

| *CDKN2A* CpG Site | Hg19 Coordinates (Chr9) | N | Min (25th, 50^th^, 75th percentile) max | Mean (sd) |
| --- | --- | --- | --- | --- |
| 1 | 21993721 | 592 | 25.2 (66.7, 72.1, 76.2) 86.2 | 69.9 (9.6) |
| 2 | 21993697 | 582 | 23.4 (65.3, 71.0, 75.3) 92.8 | 68.9 (10.0) |
| 3 | 21993694 | 496 | 18.3 (47.0, 53.3, 58.4) 65.6 | 51.8 (8.5) |
| 4 | 21993654 | 658 | 20.9 (66.8, 72.7, 77.6) 94.5 | 71.2 (10.1) |
| 5 | 21993645 | 643 | 17.6 (54.9, 60.5, 66.0) 84.2 | 59.3 (10.0) |
| 6 | 21993638 | 643 | 25.1 (66.4, 72.5, 77.1) 90.9 | 70.4 (10.1) |
| 7 | 21993629 | 601 | 17.1 (55.5, 61.4, 67.2) 80.3 | 60.5 (9.5) |
| 8 | 21993603 | 632 | 23.6 (69.9, 75.0, 79.0) 99.6 | 73.0 (9.6) |
| 9 | 21993583 | 546 | 22.0 (58.8, 65.1, 74.3) 85.6 | 65.3 (10.4) |

**Online Supplementary Figure 1. CpG Clustering. Spearman correlation of methylation levels at CpGs 1-9 within the CDKN2A region.** Four distinct clusters are defined: 1-2, 3, 4-8 and 9. (A) Correlations in the SWS cohort. (B) Median absolute deviation (MAD) scores within the 4 clusters in SWS Cohort.

(A) SWS cohort correlations

(B) SWS cohort median absolute deviations (MAD) scores

**Online Supplementary Table 2:** Sequences of primers and probes used for PCR of bisulfite converted DNA, cloning, EMSAs, and siRNA work.

| Pyrosequencing Primer | Sequence |
| --- | --- |
| CDKN2A 1-3 F | AGTAGGAAAGGTGTATTTTAAGTATATTT |
| CDKN2A 1-3 S | AGAATTATTGTTAATTATTTAAGTT |
| CDKN2A 4-9 F | TGGGGAGAATTATTGTTAATTATTTAAGTT |
| CDKN2A 4-9 S | TAGGAGAGTGGAGGA |
| CDKN2A 8-9 S | GTAGGTAGAGATTTTTTGAAATGT |
| CDKN2A 1-3 R Bio | TATCTCACCAATCCTCCACTCTCCTAAA |
| CDKN2A 4-9 R Bio | AAAAACCCATTTCCCTATTAACTACA |
| Cloning Primer | **Sequence** |
| ANRIL F1 | TTGGGTACCACCTCTAACTCACAAAGAAAGC |
| ANRIL R1 | GCCAAGCTTTGGGAATGACTAAGACACAC |
| ANRIL F2 | GAGAAGCTTCCCAGGATATTCGGGACTCA |
| ANRIL R2 | TTACCATGGTGTCAGGTGACGGATGTAGC |
| EMSA Primer | **Sequence** |
| CDK EMSA 1F | TTTCTGGAGGCGGCCTTTTTTC |
| CDK EMSA 2,3F | CCCCAGCCTCCCGGCGGGGTCACCC |
| CDK EMSA 4-7F | GAGTGGAGGACCCGTGAGATACGGGGCACGCAGGCAGCGACTTCCTGAA |
| CDK EMSA 8, 9F | CTAACAAGGATCGTAGGATCAGTTACTGCTGCGAG |
| CDK EMSA 1R | GAAAAAAGGCCGCCTCCAGAAA |
| CDK EMSA 2,3R | GGGTGACCCCGCCGGGAGGCTGGGG |
| CDK EMSA 4-7R | TTCAGGAAGTCGCTGCCTGCGTGCCCCGTATCTCACGGGTCCTCCACTC |
| CDK EMSA 8, 9R | CTCGCAGCAGTAACTGATCCTACGATCCTTGTTAG |
| siRNA work | **Sequence** |
| Custom Silencer (Congrains et al.2013) | GAAUGUCAGUUUUGAACUAtt |

**Online Supplementary Table 3:** Relationships between methylation at CpG sites within the CDKN2A region of interest and total bone area, bone mineral content and bone mineral density in the 6 year old children (whole body minus head). Adjusted for batch effect, sex and age. β coefficients and 95% CIs have been multiplied by 10 and therefore represent the change associated with a 10% increase in methylation. p-values < 0.05 are in bold.

| CpG | Combined cohort | BA (cm^2^) | | | BMC (g) | | | BMD (g/cm^2^) | | |
| --- | --- | --- | --- | --- | --- | --- | --- | --- | --- | --- |
|  |  |  |  |  |  |  |  |  |  |  |
|  | n | β | P-value | 95% CI | β | P-value | 95% CI | β | P-value | 95% CI |
| 1-2 | 438 | -0.4251 | 0.1945 | -1.1000, 0.2179 | -6.46 | 0.0941 | (-14.02, 1.11) | -0.0004 | 0.0980 | -0.0009, 0.0001 |
| 3 | 371 | -0.1912 | 0.6477 | -1.0000, 0.6312 | -4.36 | 0.3751 | (-14.02, 5.30) | -0.0003 | 0.2826 | -0.0009, 0.0003 |
| 4-7 | 444 | -0.7167 | **0.0362** | -1.4000, -0.0465 | -11.45 | **0.0049** | (-19.42, -3.49) | -0.0007 | **0.0049** | -0.0012, -0.0002 |
| 8-9 | 398 | -0.5898 | 0.1420 | -1.4000, 0.1983 | -10.21 | **0.0342** | (-19.66, -0.77) | -0.0007 | **0.0253** | -0.0013, -0.0001 |

**Online Supplementary Table 4:** Associations between CDKN2A methylation and offspring whole body minus head BMC at 4 years in combined cohort, adjusted for child’s sex, batch effect and additionally either whole body lean mass, fat mass, or birthweight. β coefficients and 95% CIs have been multiplied by 10 and therefore represent the change associated with a 10% increase in methylation. p-values < 0.05 are in bold.

| Whole body minus head BMC (g) | | | | | | | | | | | | |
| --- | --- | --- | --- | --- | --- | --- | --- | --- | --- | --- | --- | --- |
|  | *Additionally adjusted for lean mass* | | | | *Additionally adjusted for child’s sex, fat mass* | | | | *Additionally adjusted for birthweight* | | | |
| CpG | n | β | P-value | 95% CI | n | β | P-value | 95% CI | n | β | P-value | 95% CI |
| 1-2 | 420 | -2.04 | 0.129 | (-4.67, 0.59) | 420 | -0.04 | 0.985 | (-3.93, 3.85) | 497 | -2.14 | 0.282 | (-6.05, 1.77) |
| 3 | 352 | -3.03 | 0.097 | (-6.60, 0.55) | 352 | -3.09 | 0.244 | (-8.29, 2.12) | 420 | -5.8 | **0.030** | (-11.01, -0.58) |
| 4-7 | 471 | -4.01 | **0.005** | (-6.80, -1.22) | 471 | -6.37 | **0.003** | (-10.50, -2.23) | 552 | -7.44 | **0.0004** | (-11.55, -3.33) |
| 8-9 | 405 | -4.85 | **0.003** | (-8.02, -1.69) | 405 | -7.53 | **0.002** | (-12.18, -2.89) | 475 | -8.29 | **0.0005** | (-12.91, -3.67) |

**Online Supplementary Table 5:** Associations between CDKN2A methylation and offspring whole body minus head BA, BMC and aBMD at 4 years in combined cohort, adjusted for batch effect, child’s sex, *child’s height at 4 years,* mother's LP walking speed, LP smoking, pre-pregnancy height, LP triceps skinfold thickness and parity. β coefficients and 95% CIs have been multiplied by 10 and therefore represent the change associated with a 10% increase in methylation. p-values < 0.05 are in bold.

| Whole body minus head DXA indices | | | | | | | | | | | | |
| --- | --- | --- | --- | --- | --- | --- | --- | --- | --- | --- | --- | --- |
|  | **BA (cm^2^)** | | | | **BMC (g)** | | | | **aBMD (g/cm^2^)** | | | |
| CpG | n | β | P-value | 95% CI | n | β | P-value | 95% CI | n | β | P-value | 95% CI |
| 1-2 | 484 | -1.23 | 0.428 | (-4.27, 1.81) | 484 | -2.31 | 0.079 | (-4.89, 0.27) | 484 | -0.002 | 0.10 | (-0.0044, 0.0004) |
| 3 | 408 | -2.59 | 0.220 | (-6.73, 1.55) | 408 | -3.67 | 0.041 | (-7.19, -0.15) | 408 | -0.003 | 0.074 | (-0.0063, 0.0003) |
| 4-7 | 538 | -2.75 | 0.089 | (-5.92, 0.42) | 538 | -3.9 | 0.005 | (-6.60, -1.20) | 538 | -0.003 | 0.021 | (-0.0055, -0.0004) |
| 8-9 | 461 | -5.2 | 0.003 | (-8.67, -1.73) | 524 | -4.02 | 0.0002 | (-6.48, -1.56) | 461 | -0.0039 | 0.008 | (-0.0069, -0.0010) |

**Online Supplementary Table 6:** Associations between CDKN2A methylation and offspring whole body minus head BA, BMC and aBMD at 4 years in combined cohort, adjusted for batch effect, child’s sex, *child’s weight at 4 years,* mother's LP walking speed, LP smoking, pre-pregnancy height, LP triceps skinfold thickness and parity. β coefficients and 95% CIs have been multiplied by 10 and therefore represent the change associated with a 10% increase in methylation. p-values < 0.05 are in bold.

| Whole body minus head DXA indices | | | | | | | | | | | | |
| --- | --- | --- | --- | --- | --- | --- | --- | --- | --- | --- | --- | --- |
|  | **BA (cm^2^)** | | | | **BMC (g)** | | | | **aBMD (g/cm^2^)** | | | |
| CpG | n | β | P-value | 95% CI | n | β | P-value | 95% CI | n | β | P-value | 95% CI |
| 1-2 | 482 | 1.05 | 0.536 | (-2.27, 4.37) | 482 | 0.77 | 0.539 | (-1.69, 3.24) | 482 | 0.0005 | 0.632 | (-0.0016, 0.0026) |
| 3 | 407 | -0.29 | 0.900 | (-4.86, 4.27) | 407 | -0.49 | 0.778 | (-3.87, 2.90) | 407 | -0.0003 | 0.818 | (-0.0032, 0.0026) |
| 4-7 | 536 | -1.24 | 0.485 | (-4.73, 2.25) | 536 | -1.54 | 0.257 | (-4.20, 1.13) | 536 | -0.0009 | 0.448 | (-0.0032, 0.0014) |
| 8-9 | 459 | -2.89 | 0.144 | (-6.78, 0.99) | 459 | -2.54 | 0.103 | (-5.59, 0.51) | 459 | -0.0011 | 0.429 | ( -0.0038, 0.0016) |

**Online Supplementary Table 7:** Linear regression of CDKN2A CpG sites 1-9 on 4 year DXA measurements (bone area, bone mineral density, bone mineral content) on SWS Combined cohort, adjusted for sex, SWS batch effect, LP walk speed, LP triceps skinfold thickness, smoking during late pregnancy, mother's pre-pregnancy height, and parity. Regression coefficients (and 95% CIs) have been multiplied by 10, and therefore represent the average change in bone outcome per 10% change in DNA methylation. q-values were obtained using the Simes method, and highlighted bold if <0.05.

| Whole body minus head DXA indices | | | | | | | | | | | | | | | |
| --- | --- | --- | --- | --- | --- | --- | --- | --- | --- | --- | --- | --- | --- | --- | --- |
|  | **BA (cm^2^)** | | | | | **BMC (g)** | | | | | **aBMD (g/cm^2^)** | | | | |
| CpG | n | β | P-value | 95% CI | q-value | n | β | P-value | 95% CI | q-value | n | β | P-value | 95% CI | q-value |
| 1 | 492 | -3.082 | 0.1507 | (-7.289, 1.125) | 0.1565 | 492 | -3.498 | 0.0829 | (-7.453, 0.457) | 0.0932 | 492 | -0.002 | 0.1441 | (-0.005, 0.001) | 0.1556 |
| 2 | 484 | -2.996 | 0.1628 | (-7.208, 1.216) | 0.1628 | 484 | -4.114 | 0.0407 | (-8.053, -0.174) | 0.0506 | 484 | -0.003 | 0.0429 | (-0.006, -0.000) | 0.0506 |
| 3 | 408 | -5.931 | 0.0431 | (-11.678, -0.184) | 0.0506 | 408 | -7.009 | 0.0099 | (-12.326, -1.692) | **0.0149** | 408 | -0.005 | 0.0154 | (-0.009, -0.001) | **0.0219** |
| 4 | 547 | -4.35 | 0.0247 | (-8.144, -0.556) | **0.0333** | 547 | -5.568 | 0.0026 | (-9.184, -1.953) | **0.0054** | 547 | -0.004 | 0.0059 | (-0.007, -0.001) | **0.0106** |
| 5 | 541 | -6.278 | 0.0016 | (-10.167, -2.388) | **0.0039** | 541 | -7.126 | 0.0002 | (-10.830, -3.421) | **0.0012** | 541 | -0.005 | 0.001 | (-0.008, -0.002) | **0.0030** |
| 6 | 541 | -5.608 | 0.0036 | (-9.370, -1.846) | **0.0069** | 541 | -7.334 | 0.0001 | (-10.906, -3.762) | **0.0012** | 541 | -0.006 | 0.0001 | (-0.009, -0.003) | **0.0012** |
| 7 | 538 | -5.853 | 0.0084 | (-10.199, -1.508) | **0.0133** | 538 | -7.168 | 0.0007 | (-11.301, -3.035) | **0.0027** | 538 | -0.005 | 0.0021 | (-0.009, -0.002) | **0.0048** |
| 8 | 524 | -5.356 | 0.0078 | (-9.297, -1.414) | **0.0132** | 524 | -6.995 | 0.0003 | (-10.752, -3.237) | **0.0015** | 524 | -0.005 | 0.0006 | (-0.008, -0.002) | **0.0027** |
| 9 | 461 | -8.261 | 0.0009 | (-13.108, -3.413) | **0.0030** | 461 | -9.106 | 0.0002 | (-13.788, -4.425) | **0.0012** | 461 | -0.006 | 0.0016 | (-0.010, -0.002) | **0.0039** |

**Online Supplementary Figure 2: Electrophoretic mobility shift assays (EMSA) examining binding within the CpG8-9 region. (A)** EMSAs with unmethylated and methylated specific probe. **(B)** transcription factor consensus sequences as competitors against unmethylated CpG8-9 probe. **(C)**  *in silico* analysis of transcription factor binding within the CDKN2A region.


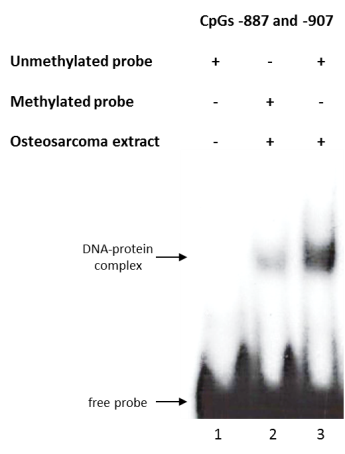

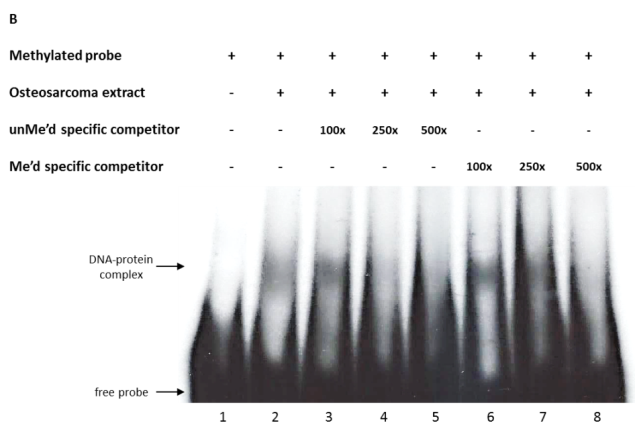
**(A)**

**(B)**


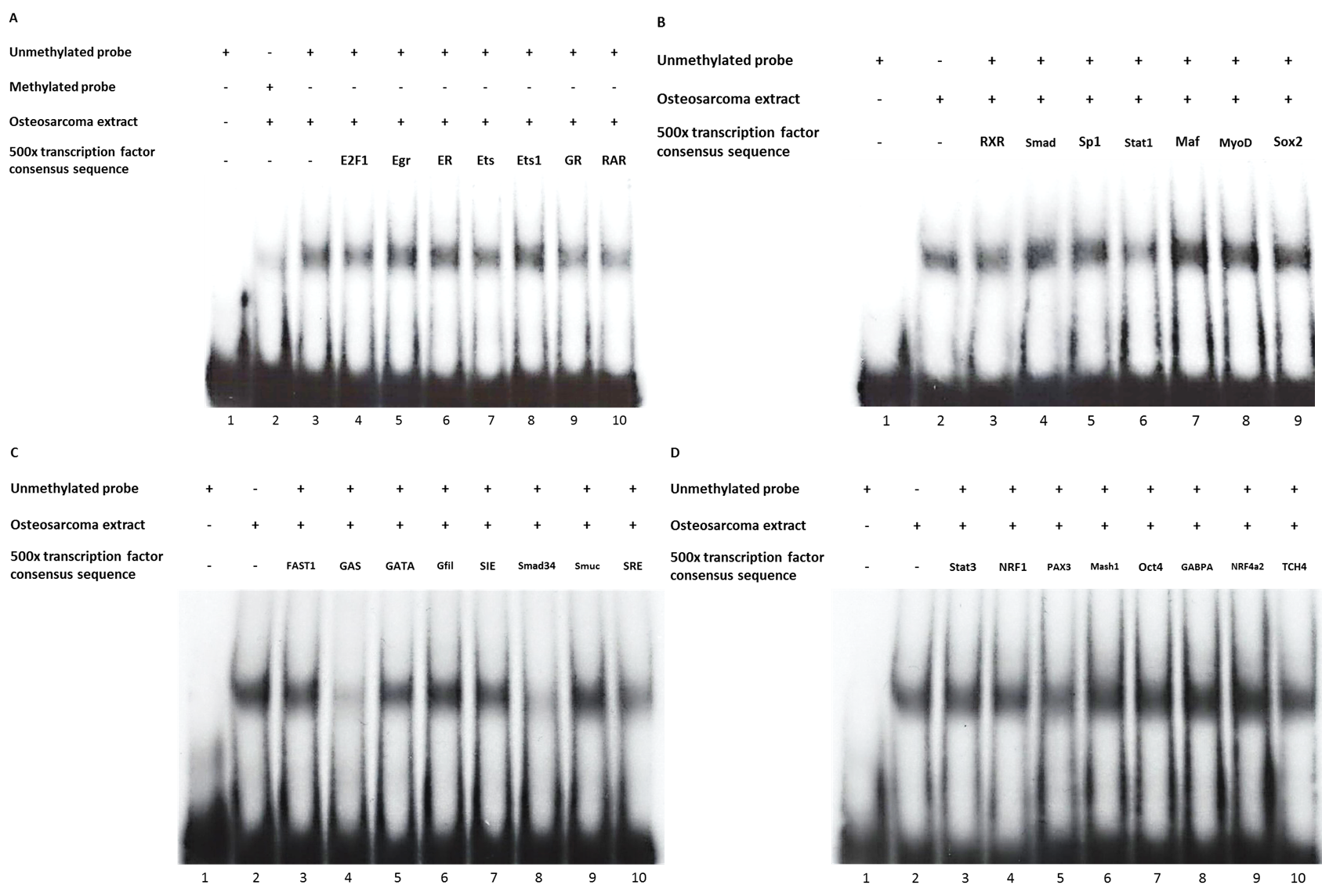


**(C)**

**References**

1. Congrains A, Kamide K, Oguro R, Yasuda O, Miyata K, Yamamoto E, Kawai T, Kusunoki H, Yamamoto H, Takeya Y, Yamamoto K, Onishi M, Sugimoto K, Katsuya T, Awata N, Ikebe K, Gondo Y, Oike Y, Ohishi M, Rakugi H 2012 Genetic variants at the 9p21 locus contribute to atherosclerosis through modulation of ANRIL and CDKN2A/B. Atherosclerosis 220(2):449-55.

2. Congrains A, Kamide K, Katsuya T, Yasuda O, Oguro R, Yamamoto K, Ohishi M, Rakugi H 2012 CVD-associated non-coding RNA, ANRIL, modulates expression of atherogenic pathways in VSMC. Biochem Biophys Res Commun 419(4):612-6.
